# Scanning Electrochemical Microscopy of Electrically Heated Wire Substrates

**DOI:** 10.3390/molecules25051169

**Published:** 2020-03-05

**Authors:** Stefan Wert, Alexander Fußstetter, Christian Iffelsberger, Frank-Michael Matysik

**Affiliations:** 1 Institute of Analytical Chemistry, Chemo- and Biosensors, University of Regensburg, Universitätsstr. 31, 93053 Regensburg, Germany; stefan.wert@chemie.uni-r.de (S.W.); alexander.fussstetter@studchem.uni-r.de (A.F.); 2 Future Energy and Innovations Laboratory, CEITEC—Central European Institute of Technology, Brno University of Technology, Purkyňova 123, 612 00 Brno, Czech Republic; christian.iffelsberger@ceitec.vutbr.cz

**Keywords:** hot-wire electrochemistry, scanning electrochemical microscopy, SECM, heated electrodes

## Abstract

We report a new configuration for enhancing the performance of scanning electrochemical microscopy (SECM) via heating of the substrate electrode. A flattened Pt microwire was employed as the substrate electrode. The substrate was heated by an alternating current (AC), resulting in an increased mass transfer between the wire surface and the bulk solution. The electrochemical response of the Pt wire during heating was investigated by means of cyclic voltammetry (CV). The open circuit potential (OCP) of the wire was recorded over time, while varied heating currents were applied to investigate the time needed for establishing steady-state conditions. Diffusion layer studies were carried out by performing probe approach curves (PACs) for various measuring modes of SECM. Finally, imaging studies of a heated substrate electrode surface, applying feedback, substrate generation/tip collection (SG/TC), and the competition mode of SECM, were performed and compared with room temperature results.

## 1. Introduction

Thermoelectrochemistry is a discipline that adds the temperature parameter to the three classical parameters of electrochemistry, known as voltage, current, and time. In a heated electrolyte solution, the resulting increased mass transport yields a higher rate of analyte conversion at the electrode surface. In such an environment, special attention is required to maintain a constant temperature around the reference electrode to prevent a shift of its potential. However, this can be neglected if heating is limited to the area closest to the working electrode, also known as non-isothermal heating. In recent research, non-isothermal heating was mostly achieved either by directly heating the electrode [1] or by irradiating the nearby solution with microwaves [2,3]. Heated electrodes have been utilized in various applications—for example, for DNA investigation [4,5,6] or in a thermoresponsive glucose biosensor [7]. Direct application of heat to electrodes was also used for electrode cleaning [8] and prevention of fouling [9]. The kinetics and heat transport processes at such electrodes were extensively studied as well [10,11,12]. In addition, the electrochemical behavior of many substances, such as nicotinamide adenine dinucleotide [8], have been characterized using heated electrodes. A convenient way of heating electrodes is the application of an alternating current (≥100 kHz) to microwires. This technique, based on the work of Gründler et al., is termed “hot-wire electrochemistry” [13,14]. Usually, gold or platinum microwires with a diameter of about 25 µm are employed as cylindrical electrodes in corresponding hot-wire configurations. Due to the microwires’ low heat capacity, it is possible to change the electrode temperature very rapidly, and even beyond the boiling point of the surrounding solution for a brief moment. Hot-wire electrochemistry has been applied for trace detection of elements like arsenic [15], copper, and mercury [16]. This was also done to compare the performance of a heated wire electrode to a rotating disk electrode, with lower limits of detection achieved using the hot-wire setup. The effects of convection and temperature on the hybridization of DNA and RNA have been investigated as well [17]. Recently, the oxidation kinetics of acetaminophen (paracetamol) was studied, comparing the results obtained using a heated microwire in a cooled electrolyte versus using a rotating disk electrode in heated solution [18]. Further improvements to the hot-wire setup have been achieved by modifying the surface of gold microwires depositing carbon nanotubes, which resulted in increased reversibility of voltammetric experiments [19].

For the electrochemical characterization of surfaces, scanning electrochemical microscopy (SECM) has become an established technique [20,21,22,23]. Its key component is an ultramicroelectrode (UME), which is scanned across a surface or interface of interest. A variety of measurement modes can be applied, with the most common ones being the feedback, substrate generation/tip collection (SG/TC), and competition modes. In feedback mode, measurements are carried out in a solution containing a redox-active mediator species while a potential is applied at the UME, resulting in conversion of the mediator at a rate that is dependent on the electrochemical activity and topography of the surface of interest. In SG/TC mode, the species converted at the probe is generated at the substrate, with no detectable species present in the bulk solution, resulting in a lower background current. This is a frequently applied measuring mode, e.g., to study corrosion processes [24,25,26]. In competition mode, the same potential is applied to probe and substrate, enabling the investigation of the catalytic activity of surfaces [27]. Both SG/TC and competition mode suffer from the limitation of a growing diffusion layer at macroscopic substrates over time, resulting in poor reproducibility of repeated measurements. To overcome this problem, our group has introduced forced convection into the SECM cell, either by stirring [28] or by a flow of the electrolyte solution [29], resulting in a rapidly established steady-state diffusion layer. In addition, forced convection led to a higher contrast in feedback mode measurements [30].

Since heating the electrolyte results in an increased convection rate, the non-isothermal approach increases mass transport exclusively near the heated electrode. Boika et al. have made use of this effect by employing heated UMEs as probes in SECM. They refer to this as “hot tip SECM” [31,32]. Other groups have investigated the impact of heated samples, showing that temperature impacts the contrast between conductive/non-conductive areas in feedback mode [33], as well as the possibility of altering the activity of immobilized enzymes on surfaces during SECM studies [34]. Numerical simulations have enabled the estimation of convective effects in SECM measurements resulting from temperature differences between the electrolyte and the surrounding environment [35]. The open circuit potential (OCP) of a UME can be used to determine the local temperature at the electrode/electrolyte interface [36]. Another method was described by using a thermocouple probe in conjunction with SECM [37].

In this work, we present a novel hot-wire electrochemistry setup, featuring flattened microwires suitable for SECM studies under well-defined substrate temperature conditions. The steady-state voltammetric behavior under heat application is shown. For several measuring modes of SECM, approach curves of heated wire substrates have been studied, and imaging experiments have been carried out.

## 2. Results and Discussion

**Figure 1:**
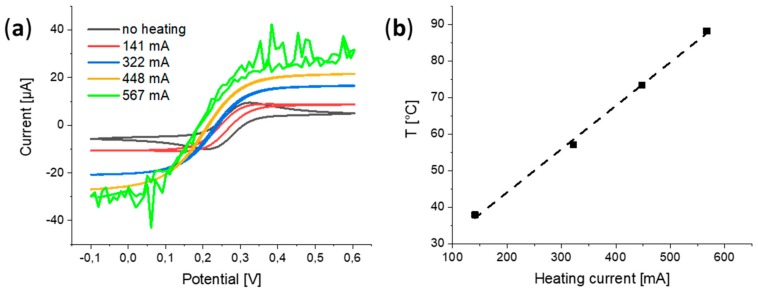
(**a**) Cyclic voltammograms (CVs) of a flattened Pt wire (width, 70 µm) in 5/5 mM ferro/ferricyanide solution with 1.0 M KNO_3_ as supporting electrolyte. The applied alternating current (AC) at the wire varied between the CVs. Scan rate: 100 mV/s. (**b**) Plot of heating currents applied at the wire during CVs and their respective temperatures, obtained from half-wave potentials.

The effect of heating the wire electrode on cyclic voltammograms (CVs) was studies in 5 mM ferro/ferricyanide by applying different magnitudes of alternating current (AC). Figure 1a shows CVs recorded while different heating currents were applied. The resistance of the flattened wire (width = 70 µm) substrate at room temperature was 5.8 Ω. Without heating, a CV with the typical shape for a quasi-reversible, one-electron transfer reaction with significant contribution of planar diffusion was obtained (black curve). Upon applying increasing heating currents, steady-state voltammetric responses could be established. This trend corresponded well to the effects observed when forced convection is introduced to macroelectrodes or when decreasing the electrode size down to dimensions for an UME. Disturbances in the voltammetric response started to occur when the wire was heated close to the boiling point of the electrolyte solution, causing the formation of bubbles at the electrode surface (green curve in Figure 1a). The in situ heating of the wire induced a potential shift towards lower potentials. The half-wave potentials of the recorded CVs could be used to determine the temperature of the wire/solution interface, shown in Figure 1b. Based on this, it was found that a temperature of 57.4 °C (corresponding to a heating current of 322 mA) was sufficient to eliminate the hysteresis of the CVs of the investigated electrochemical system. A linear correlation between heating current and wire/solution interface temperature could be observed as well.

**Figure 2:**
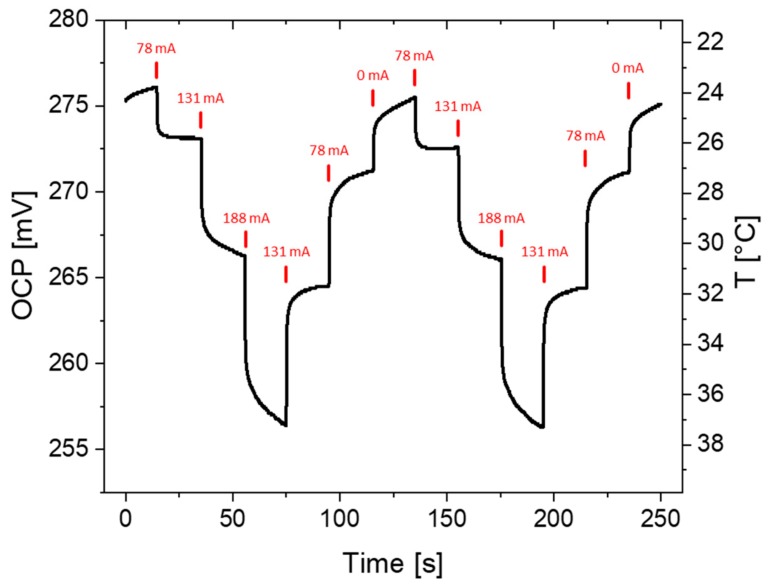
Open circuit potential (OCP) measured over time at a flattened wire substrate in a solution of 5/5 mM ferro/ferricyanide and 1.0 M KCl. The applied AC was varied in time intervals of 20 s (indicated by red marks), and the change in OCP with the corresponding temperature was monitored.

In the following section, the substrate electrode was studied in a physiologically relevant temperature region. For the investigation of temperature changes directly at the substrate, the OCP dependent on the applied heating AC was measured, which is in direct correlation with the temperature. Figure 2 shows the OCP measured at a hot-wire substrate over the course of 250 s, while the heating AC was changed every 20 s. Initially, no heating current was applied, and a temperature of 24 °C was measured. Upon increasing the heating current stepwise to 188 mA, the temperature reached values above 37 °C. However, the time required to establish a stable OCP increased with larger heating steps. For example, a stable OCP was observed after less than 10 s when increasing the AC from 0 to 78 mA, whereas stabilization took longer with increasing heating currents. The opposite effect was observed when decreasing the heating AC stepwise. As the applied AC was decreased from 188 to 131 mA, the temperature stabilized at 32 °C within 20 s. Lowering the AC further led to a prolonged time being needed to establish a constant temperature at the wire, since the heated glass substrate under the wire prevented it from cooling down. Quick temperature adjustments can be done at the wire within seconds; however, larger changes require more time.

**Figure 3:**
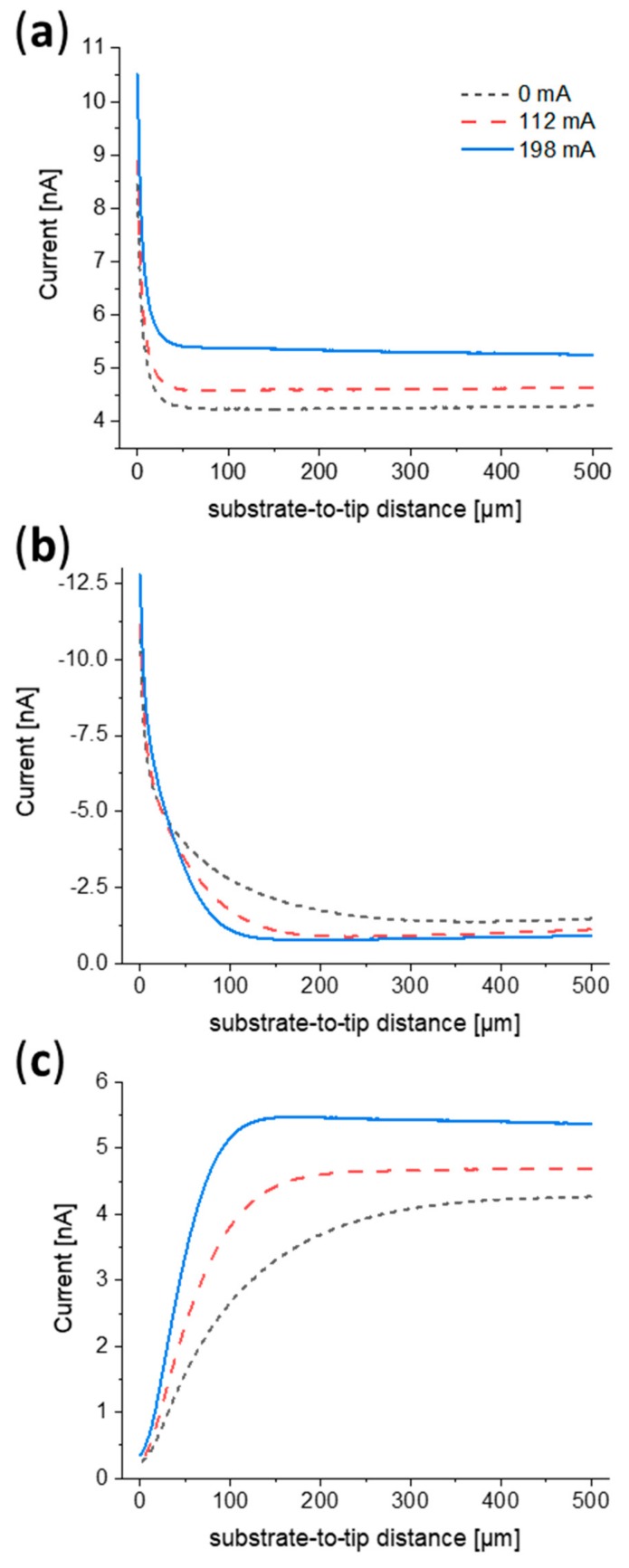
Probe approach curves (PACs) with a 25 µm probe towards a flattened Pt wire in different scanning electrochemical microscopy (SECM) operation modes for different heating currents flowing through the wire. Measurements were carried out in 1.5 mM FcMeOH and 200 mM KNO_3_. (**a**) Feedback mode: E (probe) = 0.5 V. (**b**) SG/TC mode: E (probe) = 0 V, E (wire) = 0.5 V. (**c**) Competition mode: E (probe) = E (wire) = 0.5 V.

The influence of different temperatures of the flattened wire substrate on the approach response of probes in different SECM operational modes was investigated in a solution of ferrocenemethanol (FcMeOH). Figure 3 illustrates probe approach curves (PACs) recorded in different measuring modes while heating ACs of different magnitude were applied at the wire. In feedback mode (Figure 3a), the substrate-to-tip region where the feedback response started to occur remained the same for all temperatures investigated. An overall increase of current was observed when the wire was heated, with a greater impact close to the substrate, since a vertical temperature gradient was established. In SG/TC mode (Figure 3b), heating influenced the diffusion layer around the wire. At higher temperatures, currents started to increase at shorter substrate-to-tip distances, as the diffusion layer thickness tended to decrease. The thinner diffusion layer resulted in higher currents close to the wire, in contrast to lower currents at larger distances. The occurrence of a shrunken diffusion layer at higher temperatures was observed in the competition mode approach curves (Figure 3c) as well. Within the diffusion layer, currents decreased as the probe approached the substrate, since the latter consumed most of the FcMeOH. At large substrate-to-tip distances, higher currents were measured when heating was applied, since convection was increased and thus more mediator reached the active area of the UME. In addition, in the region out of feedback or diffusion layer range, currents slightly increase in the feedback and competition mode approach curves (Figure 3a,c) when the wire was approached. This indicated the presence of a temperature gradient connected with a higher rate of mass transport closer to the wire.

**Figure 4:**
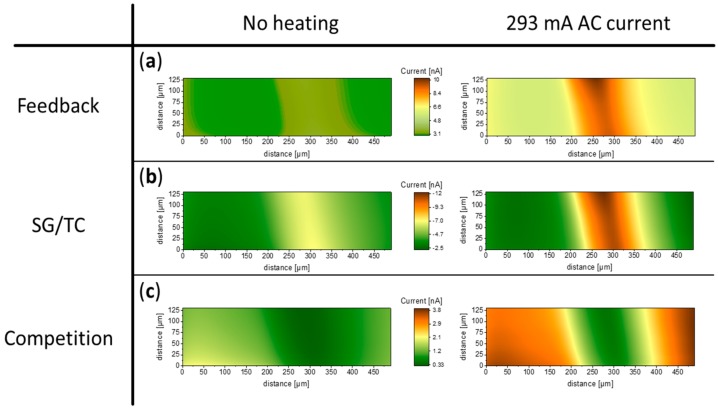
Images of a flattened Pt wire (width = 70 µm) recorded in different SECM measuring modes, with a 25 µm probe without heating (left side) and with heating (right side). (**a**) Images recorded in feedback mode; (**b**) and (**c**) were obtained in SG/TC and competition mode, respectively. E (Probe) = 0.5 V (feedback and competition mode), 0 V (SG/TC mode); E (wire) = 0.5 V (SG/TC and competition mode); scan rate: 100 µm/s, quiet time 20 s. The probe was approached to a tip-to-wire distance of 7 µm.

The effect of heating of a flattened wire on corresponding SECM images studies in different operational modes is shown in Figure 4. All measurements were done in the constant-height mode. In feedback mode (Figure 4a), heating yielded significantly higher currents above both the wire and glass surface, due to increased mass transport following the temperature gradient. The current increase above the latter might be a result of heat transfer between the hot wire and the glass substrate, as they were in direct contact. Nevertheless, a significant improvement of contrast was observable under heated conditions, also corresponding well to the feedback approach curves discussed earlier. As the disk diameter of the active probe area is in the same range as the substrate dimension, the images show an enlarged width of the substrate. In the SG/TC mode images (Figure 4b), drastically increased currents above the wire were observed, while no significant change in the glass region was noticeable upon applying heat. As a result, an image exhibiting higher contrast was obtained. In addition, a more compact and stable diffusion layer around the wire was established by convective effects introduced by heating, leading to a sharper contrast. In competition mode (Figure 4c), the background current increased with heating, while the current near the wire remained at a comparable range as was found without heating. The image recorded with heating shows a much better resolution. This observation could be explained by the formation of a stable and compact diffusion layer around the wire, similar as in the SG/TC image with heating.

## 3. Materials and Methods

### 3.1. Chemicals

For SECM imaging, an aqueous solution of 1.5 mM ferrocenemethanol (FcMeOH, 99%; ABCR, Karlsruhe, Germany) and 0.2 M KNO_3_ (Merck KGaA, Darmstadt, Germany) was used as a mediator solution. All other experiments were carried out in an aqueous solution containing 5 mM of both K_3_(Fe(CN)_6_) and K_4_(Fe(CN)_6_), as well as 1 M KNO_3_. All solutions were prepared using ultrapure water with a resistivity higher than 18 MΩ cm^−1^ (membraPure, Bodenheim, Germany).

### 3.2. Instrumentation

**Figure 5:**
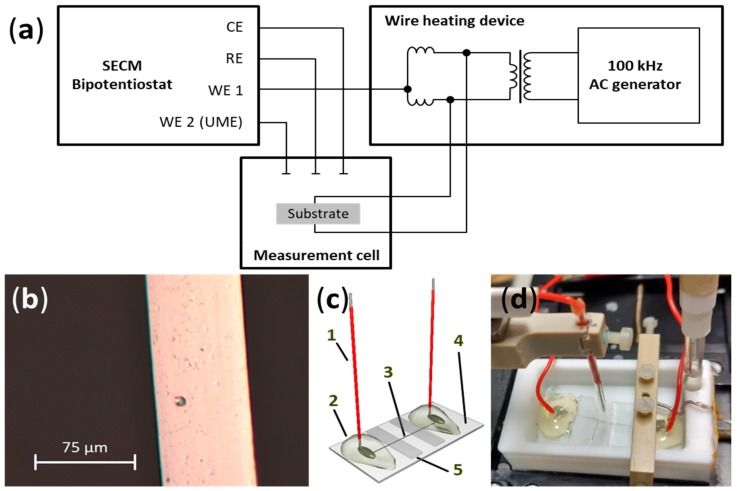
(**a**) Schematic representation of the experimental setup, showing the electrical connections between the SECM bipotentiostat, AC generator, and measurement cell (scheme adapted from [13]). (**b**) Micrograph of a flattened Pt wire. (**c**) Scheme of a hot-wire substrate used in experiments. 1: Copper cables soldered to the Pt wire. 2: Hot glue for insulation of electrical contacts. 3: Flattened Pt wire. 4: Photoresist-covered glass sheet. 5: Glass sheets for wire stabilization. (**d**) Image of the measurement cell used for SECM measurements, containing electrodes and hot-wire substrate.

The setup used for all experiments consisted of a commercial SECM (CHI 920C, CH Instruments, Austin, United States), with an electrochemical cell made of polytetrafluoroethylene and a wire heating device, which was used to apply a 100 kHz AC with varying amplitude to Pt wires. Heating intensity could be adjusted by an arbitrary percentage value corresponding to a certain voltage amplitude applied. The heating device was laboratory-built, and mainly consisted of a radio frequency generator and a radio frequency transformer, based on the constructions proposed by Gründler [13]. The working electrode channel of the bipotentiostat, which was used for measurements directly at the heated wire, was connected to the wire heating device, enabling voltammetric measurements without interference from heating AC. A scheme of the configuration is shown in Figure 5a.

For the fabrication of flattened microwires, a 3 cm piece of Pt wire with a diameter of 25 µm (Goodfellow GmbH, Hamburg, Germany) was placed between two glass slides, and force was applied using a hammer. To prevent breaking of the glass, the glass slides were placed between polymethylmethacrylate plates. When a flattened wire with a diameter of 70 µm (Figure 5b) was obtained, it was fixed on a glass slide below two additional sheets of glass to prevent wire movement, leaving about 5 mm of blank wire between them. These sheets were adhered using SU8.5 photoresist (Microchem Corp., Westborough, United States). Hardening of the photoresist was achieved by heating the substrate (95 °C, 5 min) in a convection oven, exposing it to UV light (365 nm, 0.2 W, 30 s), followed by another heating step (95 °C, 5 min). Prior to establishing electrical connections at both ends of the wire, a layer of SU8.5 was applied to the remaining glass substrate, except for the area between the glass sheets, and was then processed as before. This was done to improve the adhesion of hot glue and soldering tin applied later. Both ends of the wire were soldered onto the substrate, together with copper wires (0.5 mm inner diameter, 1.1 mm outer diameter, Pollin Electronic GmbH, Pförring, Germany) that had 5 mm of insulation removed at either end. The soldering joints were sealed by applying hot glue (Pattex, Henkel AG & Co. KGaA, Düsseldorf, Germany). Figure 5c shows a finished substrate. It was fixed in the electrochemical cell using a lab-constructed polyether ether ketone sample holder (Figure 5d).

Experiments were performed in an electrochemical cell containing redox mediator solution, with a Pt wire as counter electrode and an Ag/AgCl 3M KCl reference electrode immersed into the solution. All potentials mentioned herein refer to this reference system.

Voltammetric studies of the substrate were conducted in a solution of ferro/ferricyanide. The heating current was varied to determine the limit where experiments could be performed. Three consecutive CVs were recorded for each AC current investigated. In addition, chrono-OCP measurements were performed while the heating was varied between 0 and 188 mA, to test the possibility of undergoing quick temperature changes. Substrates were cleaned with isopropanol and water before measurements. Temperature calibration was done in an electrochemical cell connected to Peltier elements (KIMILAR, TEC1-12706). An external current was applied to the Peltier elements to establish different temperatures, and three consecutive CVs were recorded. The corresponding temperatures, measured with a thermometer, could then be related to the respective half-wave potentials. The temperature calibration is included in the Supporting Information S1.

PACs were carried out using a UME with an electrode diameter of 25 µm and an RG value of 4. This value represents the ratio between the total probe tip diameter and the diameter of the electroactive area. Fabrication of probes was done according to a procedure described elsewhere [38]. The center of the flattened wire was approached in feedback mode in an unheated state until a current corresponding to 200% of the current measured in the bulk solution was obtained. The approach was then repeated as the wire was heated, travelling the same distance as was done in the PACs without heating. For approaches in feedback mode, a potential of 0.5 V was applied at the UME, while the substrate remained at OCP. In both SG/TC and competition mode, a substrate potential of 0.5 V was set, with a probe potential of 0.0 V and 0.5 V, respectively.

SECM images in feedback, SG/TC, and competition modes were recorded with and without heating. An area of 135 × 500 µm covering the wire was imaged at a tip-to-wire distance of 7 µm. Measurements were performed with 1.5 mM FcMeOH as redox mediator, with an E (Probe) and E (wire) of 0.5 V and OCP, respectively (feedback mode); and 0.0 V and 0.5 V, respectively (SG/TC mode); as well as 0.5 V for both tip and wire (competition mode). The pixel size was set to 10 µm with a probe velocity of 100 µm/s. Heating was applied 10 min prior to imaging, to ensure steady temperatures during measurements.

## 4. Conclusions

A new hot-wire electrochemistry setup suitable for SECM studies was developed and characterized. In contrast to cylindrical microwires used in conventional hot-wire electrochemistry, flattened microwires were implemented in a planar glass substrate, enabling a straightforward operation in SECM measurements.

The new setup was successfully characterized via CV and OCP measurements, as well as both PACs and images of the microwire substrate recorded in feedback, SG/TC, and competition modes of SECM. CVs revealed that mass transport between wires and bulk solution drastically increased with a higher wire temperature. In this context, the present hot-wire SECM configuration can be seen as a new option of hydrodynamic SECM. Fast changes of temperature could be carried out, shown by OCP monitoring. Finally, SECM images of the heated microwire exhibited higher contrast in comparison to images obtained at room temperature. The concept presented in this manuscript paves the way for a wide range of studies of temperature-dependent processes by means of SECM.

## Supplementary Materials

The following are available online, Figure S1: Temperature calibration
