# Supplementary material for: Scanning Electrochemical Microscopy of Electrically Heated Wire Substrates
Source: Molecules. 2020 Mar 5;25(5):1169. doi: 10.3390/molecules25051169 (PMC7179101; doi:10.3390/molecules25051169)
Supplement: Supplementary file 1 [file molecules-25-01169-s001.pdf]

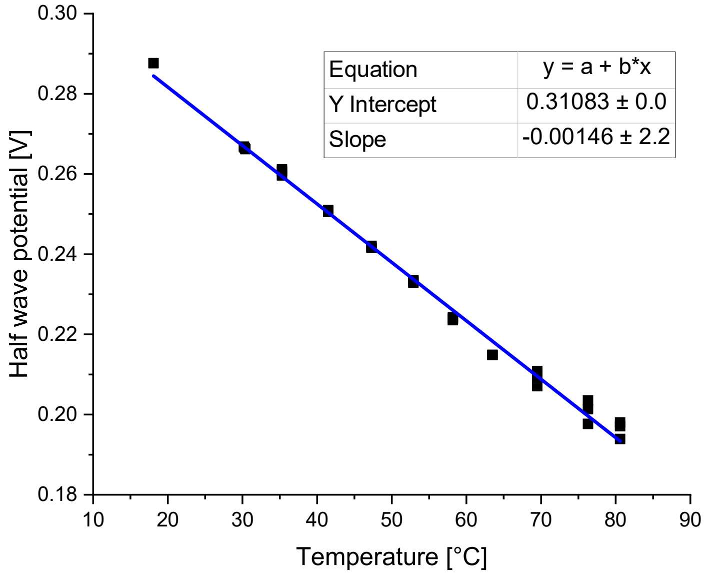

Figure S1: Temperature calibration obtained by recording CVs three times consecutively for each temperature investigated and plotting the respective half wave potentials. Measurements were performed after a constant temperature was established and conducted in an electrochemical cell connected to Peltier elements for temperature control. As working electrode, a 25 µm Pt disk electrode was utilized and potentials were measured versus a Ag/AgCl 3M KCl reference system.
